# Atypical Chest Pain Leading to the Diagnosis of Acute Promyelocytic Leukemia: A Case Report

**DOI:** 10.7759/cureus.77196

**Published:** 2025-01-09

**Authors:** Roy L Dennis, Dade Dorsey, Mitchell Scott, Ahmed Shaban, Seraphim Himes

**Affiliations:** 1 Internal Medicine, Oklahoma State University Medical Center, Tulsa, USA; 2 Internal Medicine, Edward Via College of Osteopathic Medicine (VCOM) Carolinas, Spartanburg, USA; 3 Emergency Medicine, Oklahoma State University Medical Center, Tulsa, USA; 4 Emergency Medicine, Oklahoma State University College of Osteopathic Medicine, Tulsa, USA

**Keywords:** acute coronary syndrome, acute promyelocytic leukemia (apml), hemato-oncology, leukocytosis, thrombocytopenia

## Abstract

Acute promyelocytic leukemia (APML) is a rare leukemia that leads to complications of renal toxicity, infections, leukocytosis, hemorrhaging, and disseminated intravascular coagulation, which is fatal. APML normally presents with bruising, bleeding, weakness, and infections. Patients can present with chest pain and shortness of breath due to coagulopathy. The workup for APML usually occurs when patients' labs return with leukocytosis and thrombocytopenia. Here, we report a case of a 75-year-old female with a past medical history of bronchiectasis, hypertension, and rheumatic fever who presented with chest pain and shortness of breath with elevated troponins and a normal EKG. The patient had worsened thrombocytopenia, leukocytosis, and an elevated D-dimer that did not resolve with steroids. The following report presents a patient diagnosed with APML with the initial symptoms of chest pain and shortness of breath without signs of acute coronary syndrome, leading to the diagnosis and treatment with ATRA.

## Introduction

Acute promyelocytic leukemia (APML) is a variation of acute myelocytic leukemia (AML) in which leukemic cells are halted at a distinct stage in cellular maturation, specifically the promyelocyte stage [1]. APML accounts for 5% to 20% of all cases of AML, with an incidence of 1 to 2 cases per million. There are approximately 600 to 800 new cases per year in the United States. A distinct characteristic of APML is the translocation between chromosomes 15 and 17 (15;17) (q24; q21), as seen in 95% of reported APML cases [1]. This translocation causes the expression of promyelocytic leukemia (PML)-retinoic acid receptor-alpha (RARA) fusion protein, which in turn blocks differentiation and simultaneously prevents apoptosis, enabling the proliferation of leukemic progenitors [2]. Due to this proliferation, patients often have leukocytosis with pancytopenia, as the mutation causes the rapid creation of promyelocytes and decreases the production of other blood cells, such as neutrophils, red blood cells, and platelets. Patients with APML often present with complications of pancytopenia, such as weakness, fatigue, infections, and/or hemorrhagic findings. APML can exist in two forms: hypergranular and microgranular. The hypergranular form is the more dominant of the two, occurring in around 75% of all cases of APML [1,2]. This subtype is defined by promyelocytes that contain densely packed, bright pink, reddish-blue, or dark purple granules with Auer rods. The microgranular variant is defined as having a bilobed nucleus and no apparent granules on light microscopy.

While APML is not a rapidly progressing leukemia, by the time patients present with symptoms, it is usually an acute medical emergency. Due to the proliferation of white blood cells, patients with APML are at a markedly increased risk for coagulopathies such as disseminated intravascular coagulation (DIC) and primary hyperfibrinolysis. Because of its acuity, it is important to start treatment early, as up to 40% of patients may develop pulmonary or cerebrovascular hemorrhage if left untreated. While the method of coagulopathy is not completely understood, researchers have proposed several mechanisms for how this might occur. One proposed mechanism is that the rearranged RARA activates the tissue factor (TF) promoter, increasing its expression in the mutated cells. TF works by forming a complex with factor VII to activate factors XI and X, so this overexpression results in a hypercoagulable state. Another proposed mechanism is the death of cancer cells via extracellular traps (named ETosis) [3]. This cellular death pathway releases phosphatidylserine and extracellular chromatin, increasing clotting by increasing thrombin generation and fibrin formation, damaging endothelial cells and converting them to a procoagulant phenotype, and increasing plasmin generation [4]. The final proposed mechanism that causes patients to have hypercoagulability is an increase in annexin-II expression on the surface of promyelocytes [5]. Annexin II binds plasminogen and its activator, tissue plasminogen activator, increasing plasmin formation.

A potential underrecognized side effect of APML is thrombotic complications due to the patient's hypercoagulable state. While bleeding disorders are often the most common presenting symptom of APML, one study found that anywhere from 5% to 20% of patients with APML have some sort of thrombotic event as a complication of the disease, such as deep vein thrombosis, pulmonary embolism, myocardial infarction, and ischemic cerebrovascular events [6]. While it is not well understood why this occurs, it is thought that it has to do with the proposed mechanisms mentioned above.

The most common cause of death for patients with APML is a delay in treatment that leads to hemorrhage from the coagulopathies it causes. As such, APML is considered a medical emergency when there is suspicion of it. Common lab values seen during the presentation are elevated D-dimer, decreased fibrinogen, prolonged prothrombin time, partial thromboplastin time, and international normalized ratio. An elevated white blood cell count is also seen as their progenitor cell creation is increased with this disease. At the suspicion of APML, treatment with all-trans retinoic acid (ATRA) must be started as soon as possible to force cell lines to differentiate and reverse the coagulopathies.

The case outlined below represents one of the rare cases where a patient presenting with chest pain as the main symptom was found to have APML. The case underscores how a common diagnostic workup for chest pain led to the diagnosis and treatment of APML. Diagnostic workup for APML in its early stages is essential to prevent death from complications of coagulopathy and hemorrhage.

## Case presentation

Our patient was a 75-year-old female with a past medical history of rheumatic fever, pancreatitis, hypertension, and bronchiectasis. She presented with substernal chest pain that waxes and wanes but does not radiate and shortness of breath. The pain did not resolve with nitroglycerin. Prior to the patient's ED visit, the patient mentioned visiting her pulmonologist for an upper respiratory infection and was currently taking azithromycin on Mondays, Wednesdays, and Fridays while also taking daily prednisone.

Upon admission from another ED, the patient was found to have an elevated troponin of 0.102, elevated lactic acid, elevated lactate dehydrogenase, an elevated white blood count, and decreased platelets (Table 1). The patient also mentioned having right lower quadrant abdominal pain for the past month, and she had an ultrasound that showed fluid in her uterus. The patient was started on aspirin and a statin with serial EKGs that showed no ischemia. After a second attempt at nitroglycerin orally, the patient was admitted to the ICU for a nitroglycerin drip for persistent chest pain and had a peripheral blood smear.

**Table 1:** Lab values at presentation

Variables	Patient’s values	Normal lab values
White blood cells	42.8 K/cmm	4.6-12.4 K/cmm
Red blood cells	3.79 M/cmm	3.71-5.19 M/cmm
Hemoglobin	11.6 g/dL	11.9-15.0 g/dL
Hematocrit	35.7%	33.7-46.5%
Mean corpuscular volume	94.2 cmic	80.0-100.0 cmic
Platelets	46 K/cmm	150-440 K/cmm
Red cell distribution width	15.7%	10.6-14.5%
Atypical lymphocytes	37.7 K/cmm	0.8-5.0 K/cmm
Absolute monocytes	3.9 K/cmm	0.2-1.4 K/cmm
Number of red blood cells	3	0-2/100
White blood cell morphology	Normal	Normal
Anisocytosis	1+	None
Smudge cells	None	None per 100 WBCs
Macrocytosis	None	None
International normalized ratio	1.31	<1.12
Partial thromboplastin time	136.7	22.2-36.1
Prothrombin time	16.2 sec	11.9-14.6 sec
Lactate dehydrogenase	354 U/L	117-278 U/L
Lactic acid	0.8 mmol/L	0.3-2.0 mmol/L

The patient was initially started on heparin while still continuing the nitroglycerin drip. She had a CT scan without contrast that revealed no signs of pulmonary embolism; however, the scan showed right middle lobe atelectasis and bibasilar bronchiectasis. Cardiology was consulted, and the heparin and nitro drip were discontinued. The patient's echocardiogram in the ICU revealed a calculated ejection fraction of 67% with no left ventricular wall abnormalities. Cardiology recommended starting aspirin, a statin, metoprolol tartrate, and ezetimibe for atypical chest pain while having further workup for thrombocytopenia and leukocytosis inpatient. The patient's troponin downtrended to 0.08, and cardiology recommended discontinuing the nitroglycerin drip. The patient was admitted to the medicine floor.

After admission to the medicine floor, the patient presented with continuing lab abnormalities. The white blood count continued to increase to 65.6 K/cmm, with platelets decreasing to 30 K/cmm, an elevated lactate dehydrogenase of 617, and abnormal liver enzymes with an aspartate aminotransferase of 43 and an alanine aminotransferase of 28. Hematology and oncology were consulted due to the progression of the patient's thrombocytopenia and leukocytosis. The patient had a full diagnostic workup for causes of thrombocytopenia with a bone marrow biopsy, a pan CT scan to look for lymph nodes, HIV screening, a hepatitis panel, flow cytometry, fluorescence in situ hybridization (FISH) analysis, and lab values routinely monitored.

The patient had a negative viral panel and negative HIV screening. Pan CT scans revealed no evidence of lymphadenopathy but the previous bronchiectasis and nodular thickening of the adrenal gland. The CT scan of the soft tissue of the neck resulted in scattered cervical lymph nodes without pathological enlargement. Despite the negative screenings and imaging, the patient's lab values worsened. The patient's white blood cell count continued to increase over the next few days from 65.6 to 79.9 to 87.5 K/cmm, with the platelets decreasing from 26 to 20 K/cmm. Also, the D-dimer elevated acutely to above 20 ug/mL, and the fibrinogen level decreased to 146 mg/dL (Table 2). The patient was given a platelet transfusion due to decreasing platelet values.

**Table 2:** Lab values at discharge

Variables	Patient’s values	Normal lab values
White blood cells	78.6 K/cmm	4.6-12.4 K/cmm
Red blood cells	3.06 M/cmm	3.71-5.19 M/cmm
Hemoglobin	9.5 g/dL	11.9-15.0 g/dL
Hematocrit	28.5%	33.7-46.5%
Mean corpuscular volume	93 cmic	80.0-100.0 cmic
Platelets	90 K/cmm	150-440 K/cmm
Red cell distribution width	15.5%	10.6-14.5%
Atypical lymphocytes	37.7 K/cmm	0.8-5.0 K/cmm
Absolute monocytes	0.8 K/cmm	0.2-1.4 K/cmm
Number of red blood cells	1	0-2/100
Anisocytosis	1+	None
Smudge cells	Few	None per 100 WBCs
Macrocytosis	None	None
International normalized ratio	1.45	<1.12
Partial thromboplastin time	31.4	22.2-36.1
Prothrombin time	17.6 sec	11.9-14.6 sec
Fibrinogen	174 mg/dL	206-464 mg/dL
Lactate dehydrogenase	617 U/L	117-278 U/L
D-dimer	>20.0 ug/mL	≤0.48 ug/ML

With the patient's D-dimer elevating, elevated lactate dehydrogenase, elevated INR, low platelets, and prolonged prothrombin time, the medicine team started to have a concern for DIC due to APML. The medicine team wanted to start the patient on ATRA; however, the hospital where the patient was located didn’t have the treatment available. The patient was then transferred to a facility that could provide the treatment regimen.

After the patient was transferred to the facility with ATRA, the peripheral blood smear and the FISH analysis resulted. The blood smear revealed frequent blasts, and the recommendation was to continue the workup with a bone marrow biopsy and flow cytometry. The FISH analysis was performed on nuclei using a probe that hybridizes for the gene on chromosome 15q22 (Promyelocytic Leukemia SpectrumOrange) and a probe for the gene on chromosome 17q21 (Retinoic Acid Receptor 𝞪 SpectrumGreen). The FISH analysis confirmed the diagnosis of APML, which revealed the translocation t(15;17)(q22;q21) PML-RARA, which occurs in the majority of diagnoses of APML.

## Discussion

When patients present in the early stages of APML, they present with fatigue, weakness, and an immunocompromised state. Some presentations will have patients having recurrent infections, and lab work on diagnostic workups will reveal hematologic abnormalities [7]. In this case, the patient presented with an upper respiratory infection with increased airway secretions; however, chronic bronchiectasis increases the chances of upper respiratory infections. The patient, in this case, had chest pain, which led to the diagnosis of APML. The patient had chest pain with elevated troponins but no signs of ischemia. Chest pain, in our case, became an early symptom of APML that led to a diagnosis. APML causes a hypercoagulation state and increased immune responses. APML increases the risk for thromboembolic or hemorrhagic events due to the pathophysiology of expressing procoagulants and TFs on promyelocytes [8]. Also, the patient had hyperleukocytosis, which led to chest pain and dyspnea from leukostasis.

When patients present with abnormal leukocytosis and thrombocytopenia, physicians should be suspicious of hematological diseases that could be causing the patient's symptoms. The appropriate workup of peripheral blood smears and bone marrow biopsy should be initiated early on. Continued leukocytosis and thrombocytopenia warrant the next steps of flow cytometry and FISH with the markers for the gene on chromosome 15q22 and the gene on chromosome 17q21. In our case, these studies helped to confirm our suspicions of APML and the decision to start the treatment with ATRA at the outlying facility to prevent an adverse outcome [1,9]. Patients who are not diagnosed early enough in the disease prognosis could progress to the life-threatening adverse effect of DIC.

DIC is the process of overacting coagulation factors leading to blocked circulation of vascular flow or uncontrollable hemorrhaging. Untreated APML has a high risk of patients going into DIC, which is why acutely diagnosed untreated APML has a high mortality rate. When patients with APML go into DIC, they will have hypofibrinogenemia and increases in fibrinogen degradation products like D-dimer [3,10]. In our case, the key to our diagnosis was the change in the patient's D-dimer from undetectable to significantly elevated above-normal levels to above 20 ug/dL. The patient's fibrinogen significantly dropped to 146 mg/dL while still having thrombocytopenia and leukocytosis. This prompted the immediate treatment of ATRA for our patient. Also, the patient's significantly depleted platelets with a significantly high WBC increased the urgency for treatment.

Patients who have been diagnosed with APML present with leukocytosis and various stages of thrombocytopenia. The severity of the thrombocytopenia and leukocytosis determines the classification of APML into low-risk, intermediate, or severe-risk categories. Low-risk APML presents with a white blood cell count of less than 10,000 and platelet counts greater than 40,000. Intermediate APML presents with a WBC of less than 10,000 and platelets of less than 40,000. High-risk APML presents with a WBC greater than 10,000 regardless of platelets [11]. The patient, in this case, presented with high-risk APML with a WBC of 42,000 and increasing up to 87,500. The patient’s platelets decreased to 20,000, prompting platelet transfusion. This case presented a patient with high-risk APML; however, the updated treatment of ATRA as early as possible improves prognosis.

Treatment using ATRA and arsenic trioxide (ATO) in early diagnosis of APML has shown a decrease in mortality and morbidity as well as an increase in the long-term prognosis of patients. This treatment regimen has revolutionized the treatment of APML from a highly fatal disease to a treatable cancer [12]. Previous studies have looked at the cure rate of ATRA with anthracycline-based chemotherapy versus ATRA with ATO. The results showed that ATRA with ATO was the most effective treatment for APML in adolescents and older patients to induce complete remission; however, anthracycline chemotherapy-based agents may be added to treatment regimens if the patient has reached remission with ATRA and ATO.

## Conclusions

The patient was found to have APML, presenting with symptoms of chest pain and shortness of breath. Typical symptoms of APML are fatigue, hemorrhage, or easy bruising; however, the patient in this case presented with early signs of chest pain and shortness of breath with underlying bronchiectasis. The patient's persistent leukocytosis with thrombocytopenia, followed by an elevated D-dimer, led to the diagnosis of APML. When presented with unknown reasons for leukocytosis and thrombocytopenia following symptoms of atypical chest pain with shortness of breath, it is essential to have a differential of hemolytic causes.

Moreover, when presented with worsening thrombocytopenia and leukocytosis symptoms, the appropriate workup and treatment are imperative to preventing adverse events. Workup should include consultation with hematology and oncology followed by a peripheral blood smear, bone marrow biopsy, continuous lab checks, and genetic screens to rule out potential hematological diagnoses. Also, when suspecting patients with APML, treatment immediately with ATRA helps decrease morbidity and mortality. Patients should be treated before confirmation if lab values such as D-dimer are elevated with the other lab values of thrombocytopenia and leukocytosis. For patients with APML, starting treatment within the first month of onset of symptoms improves the prognosis, while not treating patients in the first month leads to a high mortality rate.
